# Correction: Origin, evolution, and success of p*bla*, the gonococcal beta-lactamase plasmid, and implications for public health

**DOI:** 10.1371/journal.ppat.1014134

**Published:** 2026-04-14

**Authors:** Tabea A. Elsener, Ana Cehovin, Connor Philp, Kate Fortney, Stanley M. Spinola, Martin C. J. Maiden, Christoph M. Tang

## Notice of Republication

This article [1] was republished on March 30, 2026, to correct an error in the author list: Stanley M. Spinola was erroneously displayed as Stanely M. Spinola. The publisher apologizes for the error.

Additionally, the raw data underlying Figs 2B-D, 3A-C, 4A-D, S2, and S5, which were not originally provided with [1], are now included with the corrected version of [1] as S1-S5 Data.

The originally published, uncorrected article and the republished, corrected article are provided here for reference.

Please download this article again to view the correct version.

## Supporting information

S1 File Originally published, uncorrected article. (PDF)

S2 File Republished, corrected article. (PDF)
