# Supplementary material for: Correction: Origin, evolution, and success of pbla, the gonococcal beta-lactamase plasmid, and implications for public health
Source: PLoS Pathog. 2026 Apr 14;22(4):e1014134. doi: 10.1371/journal.ppat.1014134 (PMC13078630; doi:10.1371/journal.ppat.1014134)
Supplement: S1 File — (PDF) [file ppat.1014134.s001.pdf]

PLOS Pathogens

RESEARCH ARTICLE

Origin, evolution, and success of p*bla*, the gonococcal beta-lactamase plasmid, and implications for public health

Tabea A. Elsener[1], Ana Cehovin[1], Connor Philp[1], Kate Fortney[2], Stanely M. Spinola[2,3,4], Martin C. J. Maiden[5], Christoph M. Tang[1]*

1 Sir William Dunn School of Pathology, University of Oxford, Oxford, United Kingdom, **2** Department of Microbiology and Immunology, Indiana University School of Medicine, Indianapolis, Indiana, United States of America, **3** Medicine, Indiana University School of Medicine, Indianapolis, Indiana, United States of America, **4** Pathology and Laboratory Medicine, Indiana University School of Medicine, Indianapolis, Indiana, United States of America, **5** Department of Biology, University of Oxford, Oxford, United Kingdom

* christoph.tang@path.ox.ac.uk

Check for updates

OPEN ACCESS

Citation: Elsener TA, Cehovin A, Philp C, Fortney K, Spinola SM, Maiden MCJ, et al. (2025) Origin, evolution, and success of p*bla*, the gonococcal beta-lactamase plasmid, and implications for public health. PLoS Pathog 21(5): e1013151. https://doi.org/10.1371/journal.ppat.1013151

Editor: H. Steven Seifert, Northwestern University Feinberg School of Medicine, UNITED STATES OF AMERICA

Received: February 11, 2025

Accepted: April 23, 2025

Published: May 6, 2025

Copyright: © 2025 Elsener et al. This is an open access article distributed under the terms of the Creative Commons Attribution License, which permits unrestricted use, distribution, and reproduction in any medium, provided the original author and source are credited.

Data availability statement: All relevant data are within the manuscript and its Supporting Information files.

Funding: o TAE is funded by the Oxford-Hoffman Graduate Scholarship, Jesus College

Abstract

Neisseria gonorrhoeae is a leading cause of sexually transmitted infection (STI) and a priority AMR pathogen. Two narrow host range plasmids, p*bla* and pConj, have contributed to ending penicillin and tetracycline therapy, respectively, and undermine current prevention strategies including doxycycline post-exposure prophylaxis (Doxy-PEP). Here, we investigated the origin and evolution of the beta-lactamase plasmid, p*bla*. We demonstrate that p*bla* was likely acquired by the gonococcus from *Haemophilus ducreyi*, and describe the subsequent evolutionary pathways taken by the three major p*bla* variants. We show that the ability of pConj to spread p*bla* promotes their co-occurrence in the gonococcal population and the spread of p*bla*. Changes that mitigate fitness costs of p*bla* and the emergence of TEM beta-lactamases that confer increased resistance have contributed to the success of p*bla*. In particular, TEM-135, which has arisen in certain p*bla* variants, increases resistance to beta-lactams and only requires one amino acid change to become an extended spectrum beta-lactamase (ESBL). The evolution of p*bla* underscores the threat of plasmid-mediated resistance to current therapeutic and preventive strategies against gonococcal infection. Given the close relationship between p*bla* and pConj, widespread use of Doxy-PEP is likely to promote spread of both plasmids, strains which carry pConj and are resistant against third generation cephalosporins, and the emergence of plasmid-mediated ESBL in the gonococcus, with significant public health consequences.

with the Sir William Dunn School of Pathology. Work in CMT's laboratory is supported by the Wellcome Trust (https://wellcome.org/, grant codes 214374/Z/18/Z and 221924/Z/20/Z). The funders had no role in study design, data collection and analysis, decision to publish, or preparation of the manuscript.

Competing interests: The authors have declared that no competing interests exist.

Author summary

Neisseria gonorrhoeae is a threat to sexual and reproductive health. With no available vaccine against gonococcal disease, control of infection depends on having effective antimicrobials for the treatment of cases and their contacts. However, strains of *N. gonorrhoeae* have acquired resistance to all classes of antibiotics, including third generation cephalosporins, the mainstay of treatment for gonococcal disease. The gonococcus can carry two resistance plasmids, the conjugative plasmid pConj and beta-lactamase plasmid p*bla*, which have rendered treatment with tetracycline/doxycycline and penicillin ineffective, respectively. Here, we investigated the origin and evolution of p*bla,* and the molecular basis of its success in the gonococcal population. We show that p*bla* was likely acquired from *Haemophilus ducreyi* and has adapted to the gonococcus both through gene loss that reduces fitness costs, and through mutations that increase resistance and favour the development of an extended spectrum beta-lactamase. Our findings show that the ability of pConj to mobilise p*bla* promotes their co-occurrence, highlighting the potential to co-select for p*bla* by favouring pConj-containing strains through doxycycline use. Our results highlight the continuing adaptation of p*bla* to its host, which could undermine treatment options in the future.

Introduction

Neisseria gonorrhoeae causes ~80 million cases of sexually transmitted infection (STIs) annually [1] and is a WHO priority pathogen due to its extensive antimicrobial resistance (AMR) [2]. *N. gonorrhoeae* has two resistance plasmids, pConj and p*bla*, which contributed to the cessation of tetracycline and penicillin therapy, respectively. Furthermore, pConj carrying *tetM* confers resistance to doxycycline as well as tetracycline, so undermines the ability of doxycycline post-exposure prophylaxis (Doxy-PEP) to prevent gonococcal infection [3,4]. pConj and p*bla* are highly prevalent in low and middle-income countries (LMICs) where syndromic treatment of STIs with doxycycline has been recommended [5,4,6]. Therefore, it is important to understand the factors driving the success of these plasmids in gonococcal populations.

pConj is a 39–42 kb conjugative plasmid, that can confer high-level tetracycline/doxycycline resistance [4,7], and can be categorised into seven variants [6]. p*bla* emerged in the gonococcus in the 1970s and encodes a TEM beta-lactamase conferring penicillin resistance [8,9]. p*bla* TEM beta-lactamases require a few amino acid changes to become an extended-spectrum beta-lactamase (ESBL) [10], which would render third-generation cephalosporins, the current first-line treatment, ineffective [11]. p*bla* is usually closely associated with pConj which can mobilise p*bla* [6,12]; around 85% of strains that harbour p*bla* also contain pConj.

There are three main p*bla* variants, characterised by the presence/absence of certain genes [5]. The 7.4 kb p*bla*.2 (also referred to as p*bla* Asia) has been considered the ancestral plasmid [13]. p*bla*.1 (5.6 kb, p*bla* Africa) is the commonest variant and

has a deletion in the replication region, while p*bla*.3 (5.1 kb, p*bla* Rio/Toronto) lacks the region implicated in p*bla* mobilisation [5,13]. Variants of p*bla* are associated with certain pConj variants and TEM alleles [5]. p*bla*.1 mostly carries TEM-1 or TEM-1_{P14S}, while p*bla*.3 is associated with TEM-135; p*bla*.2 carries TEM-1 or TEM-135. Importantly, the M182T substitution in TEM-135 is a 'stepping stone' mutation before the enzyme becomes an ESBL [10,14].

Here, we investigated the origin, evolution and characteristics of the three p*bla* variants. We demonstrate that the spread and distribution of p*bla* in gonococci results from the dynamics of its association with pConj, fitness costs, and resistance levels. p*bla* is found with mobile pConj variants and has evolved to avoid fitness costs and confer higher resistance to beta-lactams. Our results underline the threats posed by p*bla* and pConj, particularly if their spread and prevalence in the gonococcal population is promoted by the widespread implementation of Doxy-PEP.

Results

p*bla* has been acquired by the gonococcus from *Haemophilus*

*Haemophilu*s spp. are known to harbour plasmids related to p*bla* [15–20]; however, without nucleotide sequence data, these early studies could not characterise the precise relationship between *Haemophilus* and *Neisseria* beta-lactamase plasmids. Therefore, we interrogated *Haemophilus* whole genome sequences (WGS) in the PubMLST database [21] (6,896 isolates, 12 species, S1 Table) for p*bla*. We searched for Tn*2* as p*bla* TEM-1b is located on this transposon [22,23], and confirmed the presence of p*bla* replicons by searching for NEIS2960, NEIS2358 (*repA*), and NEIS2961 (*mobA*) [5]. Tn*2* is present in 12.5% of *Haemophilus influenzae* (6,403 isolates) and 19.3% *Haemphilus parainfluenzae* (269 isolates), while p*bla*-like replicons were detected in 0.3% of *H. influenzae* and 1.5% *H. parainfluenzae* isolates. However, Tn*2* and p*bla* sequences only co-occurred in two isolates of *H. influenzae* (PubMLST ids: 23482 and 33361) and *H. parainfluenzae* (PubMLST ids: 16289 and 34872).

In contrast, seven of 31 (22.6%) *H. ducreyi* isolates (S2 Table) harbour TEM-1 containing p*bla*-like plasmids. *H. ducreyi* strains can be divided into two clades/classes [24], and a p*bla.*1-like plasmid in Class I isolate and a p*bla.*2-like plasmid in a Class II isolates. The sequences of plasmids from *H. ducreyi* HD183 (Class I, 9.1 kb) and DMC64 (Class II, 10.9 kb) [25] were aligned to gonococcal p*bla*.1 and p*bla*.2, and were found to be highly similar (> 83% nucleotide identity, Fig 1A). p*bla*.1 and the 9.1 kb *H. ducreyi* plasmid carry *repB* and one copy of NEIS2964. Both the 10.9 kb *H. ducreyi* plasmid and gonococcal p*bla*.2 carry two *rep* alleles, *repA* and *repB,* as well as two copies of NEIS2964. The major difference is that Tn*2* is intact in the *H. ducreyi* plasmids while gonococcal p*bla* lack *tnpA* and have a truncated *tnpR.* These findings are consistent with p*bla* transfer from *H. ducreyi* into the gonococcus with the event associated with truncation of Tn*2*.

Two alternative scenarios can explain the occurrence of two distinct p*bla* variants in both *H. ducreyi* and *N. gonorrhoeae*: i) independent introductions of p*bla*.1 and p*bla*.2, with both introductions associated with a truncation of Tn*2*, or ii) introduction of p*bla*.2 into the gonococcus with p*bla*.1 emerging independently in *H. ducreyi* and *N. gonorrhoeae* through the deletion of *repB* and one copy of NEIS2964. Comparison of the Tn*2* deletion site in gonococcal p*bla* variants demonstrates that the plasmids carry distinct *tnpR* alleles (allele 3 and 2 for p*bla*.1 and p*bla*.2, respectively), as their truncations differ by a single nucleotide (S1A Fig), supporting the hypothesis of two independent Tn*2* truncations/introductions. We also examined the p*bla*.1 deletion site (spanning *repB* and one copy of NEIS2964). *H. ducreyi* p*bla*.2 carries two copies of NEIS2964 (alleles 2 and 3) which differ by three nucleotides (S1B Fig). Gonococcal p*bla*.2 carries alleles 1 and 2. *H. ducreyi* p*bla*.1 carries allele 3, whereas gonococcal p*bla*.1 is associated with allele 2, suggesting p*bla*.1 arose independently in *H. ducreyi* and *N. gonorrhoeae*. Therefore, analysis of Tn*2* truncations and p*bla* deletions is inconclusive about whether a single or multiple introductions of p*bla* occurred into the gonococcus.

To investigate the evolutionary relationships between gonococcal p*bla* variants further, we examined a subset of p*bla* (414 of 2,758, S3 Table) [5] with the 10.9 kb *H. ducreyi* p*bla* as reference; plasmids were from 1979-2022 with the same proportion of variants as the whole population (*i.e.,* 70% p*bla*.1, 14% p*bla*.2, 16% p*bla*.3) [5]. A maximum likelihood phylogeny placed

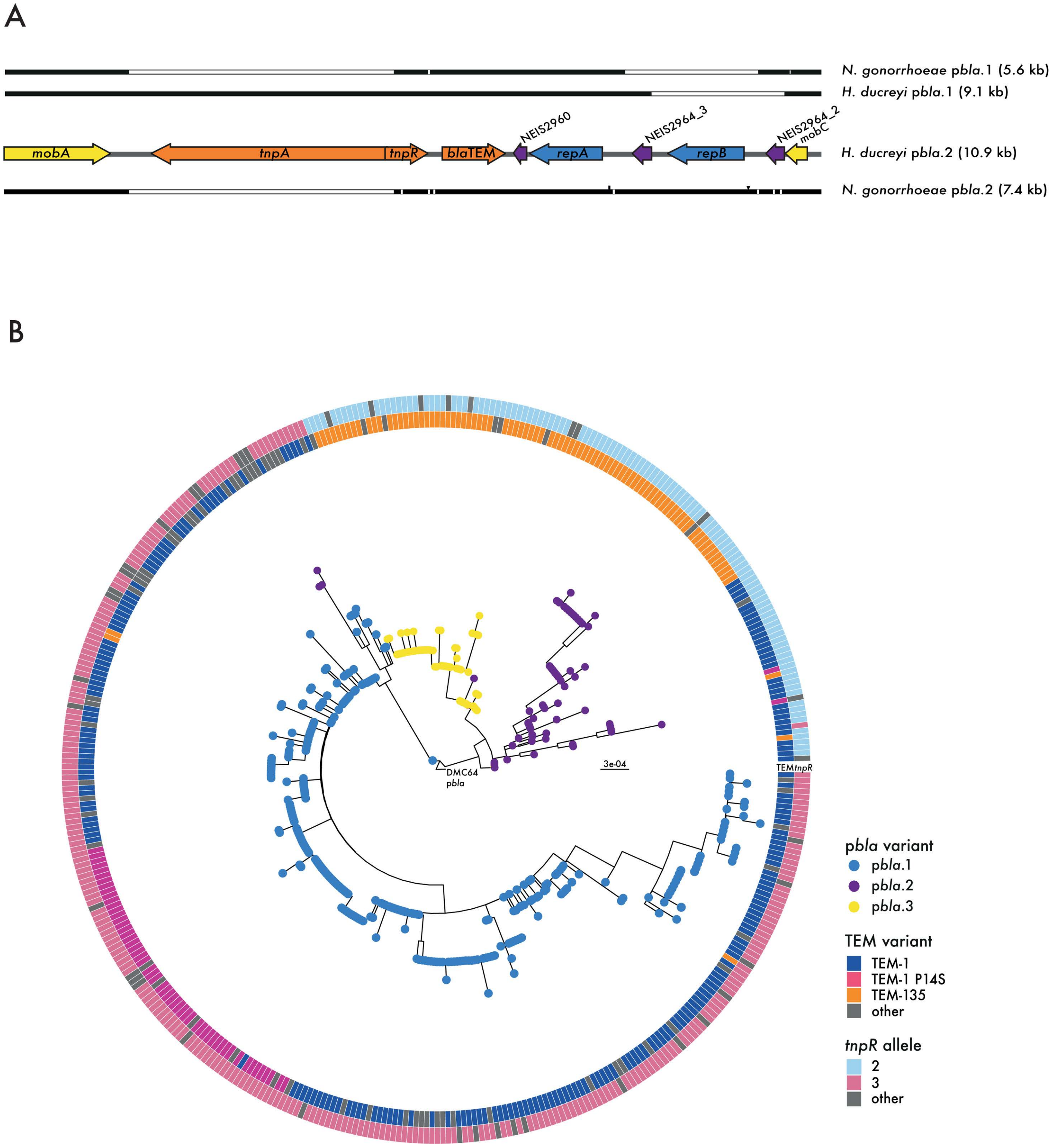

Fig 1. Evolutionary relationships between p*bla* variants. (A) Schematic representation of alignment of gonococcal and *H. ducreyi* p*bla* variants to *H. ducreyi* p*bla*.2. Aligned regions are represented as black bars, with deletion regions and nucleotide polymorphisms indicated in white within. Insertions are shown as black lines above the bars. ORFs on *H. ducreyi* p*bla*.2 are coloured according to gene function; yellow, mobilisation proteins; orange,

Tn2-derived genes including *bla*TEM; light blue, replication initiation proteins; purple, other gene function. (B) Maximum likelihood tree of 414 gonococcal p*bla* sequences with tips coloured according to p*bla* variant; the tree was rooted at *H. ducreyi* p*bla*.2. Circles indicate the *tnpR* allele and the TEM variant carried.

https://doi.org/10.1371/journal.ppat.1013151.g001

p*bla* variants into distinct clades, with p*bla*.1 split from the other variants. While *H. ducreyi* p*bla* carry TEM-1, TEM-1_{P14S} arose in p*bla*.1 while TEM-135 arose in p*bla*.2, with p*bla*.3 emerging from TEM-135 carrying p*bla*.2 (Fig 1B).

p*bla* is associated with pConj variants that promote its spread

To understand the association between p*bla* and pConj, we examined the ability of different pConj variants to transfer p*bla*. Initially, matings were performed between isogenic strains (FA1090 or 2086_K) to eliminate any barriers to horizontal gene transfer between unrelated strains [26]. Additionally, Δ*pilD* donors and recipients were constructed to block transformation [27–29], and the transfer p*bla*.1 by pConj.1, the commonest combination of these plasmids [5], was measured. p*bla*.1 was mobilised at a frequency of ~1% transconjugants/recipient for FA1090 and 2086_K (S1 Fig), while no p*bla* transfer was detected in the absence of pConj.

Conjugative plasmids can block the acquisition of other plasmids by expressing entry exclusion proteins [30]. Entry exclusion could impede p*bla* mobilisation, as the initial transfer of pConj during conjugation could block subsequent acquisition of p*bla*. pConj encodes a predicted lipoprotein annotated as TrbK entry exclusion protein [31], with 21% amino acid similarity with *Agrobacterium fabrum* Ti plasmid TrbK (locus tag: ATU_RS23180, Genbank: NC_003065.3). To examine whether pConj in a recipient can impair the transfer of p*bla*, we compared the mobilisation rates of p*bla* into pConj-free and pConj-harbouring recipients. There was no difference in p*bla* transfer into pConj-free (1.1%) and pConj-containing recipients (0.9%, Welch two-sample t-test, $p = 0.87$, S1 Fig), indicating that surface exclusion is unlikely to limit p*bla* spread.

We next evaluated p*bla* mobilisation by pConj variants that are frequently associated with p*bla* (*i.e.* pConj 1, 3, and 4, Fig 2A) and by variants that are infrequently associated with p*bla* (*i.e.* pConj.2 and 7). The conjugation frequencies of pConj.1, 3 and 4 were >79% (Fig 2B) but several orders of magnitude lower for pConj.2 and pConj.7 which are not associated with p*bla* (Fig 2A). The rate of p*bla* mobilisation correlated with conjugation frequencies (Fig 2B), with p*bla* transfer by pConj.2 undetectable in our assays (limit of detection, L.O.D. = 0.001%). Taken together, results indicate that p*bla* is associated with pConj variants that mobilise it efficiently and promote its spread in the gonococcal population.

The immobility of p*bla*.3 is reflected in its restricted distribution

There are conflicting reports about the mobility of p*bla*.3 [32–34]. Therefore, we assessed the mobilisation of wild-type p*bla* variants by pConj.1. Results indicate that wild-type p*bla*.1 and p*bla*.2 were mobilised efficiently (transfer rate, ~ 1%), while p*bla*.3 mobilisation was not detected (Fig 2C). To assess whether p*bla* variant deletions are responsible for these differences, we introduced variant-specific deletions into p*bla*.2, generating the isogenic plasmids p*bla*.1^{iso} and p*bla*.3^{iso}. Mobilisation of the isogenic plasmids did not differ from wild-type plasmids ($p = 0.82$, Fig 2C), indicating the 2.3 kb deletion in p*bla*.3 compared with p*bla*.2 is responsible for the lack of p*bla*.3 transfer. The immobility of p*bla*.3 is evident from its restricted distribution in three related lineages, whilst p*bla*.1 and p*bla*.2 are found across the gonococcal population (S3 Fig) [5].

Mobilisable plasmids deploy diverse strategies to exploit the conjugative machinery of co-existing conjugative plasmids [35]. Some mobilisable plasmids encode their own relaxase which recognises and nicks their origin of transfer (*oriT*) and then guides the plasmid DNA through the Type 4 secretion system encoded by the conjugative plasmid. Alternatively, the *oriT* of mobilizable plasmids can be recognised by the relaxase encoded by a conjugative plasmid. Therefore, we next examined the genes responsible for the immobility of p*bla*.3 by generating isogenic p*bla*.2 mutants lacking genes absent in p*bla*.3, and assessing their ability to be mobilised between *pilD* mutants of FA1090 by pConj1. The p*bla*.3-characteristic

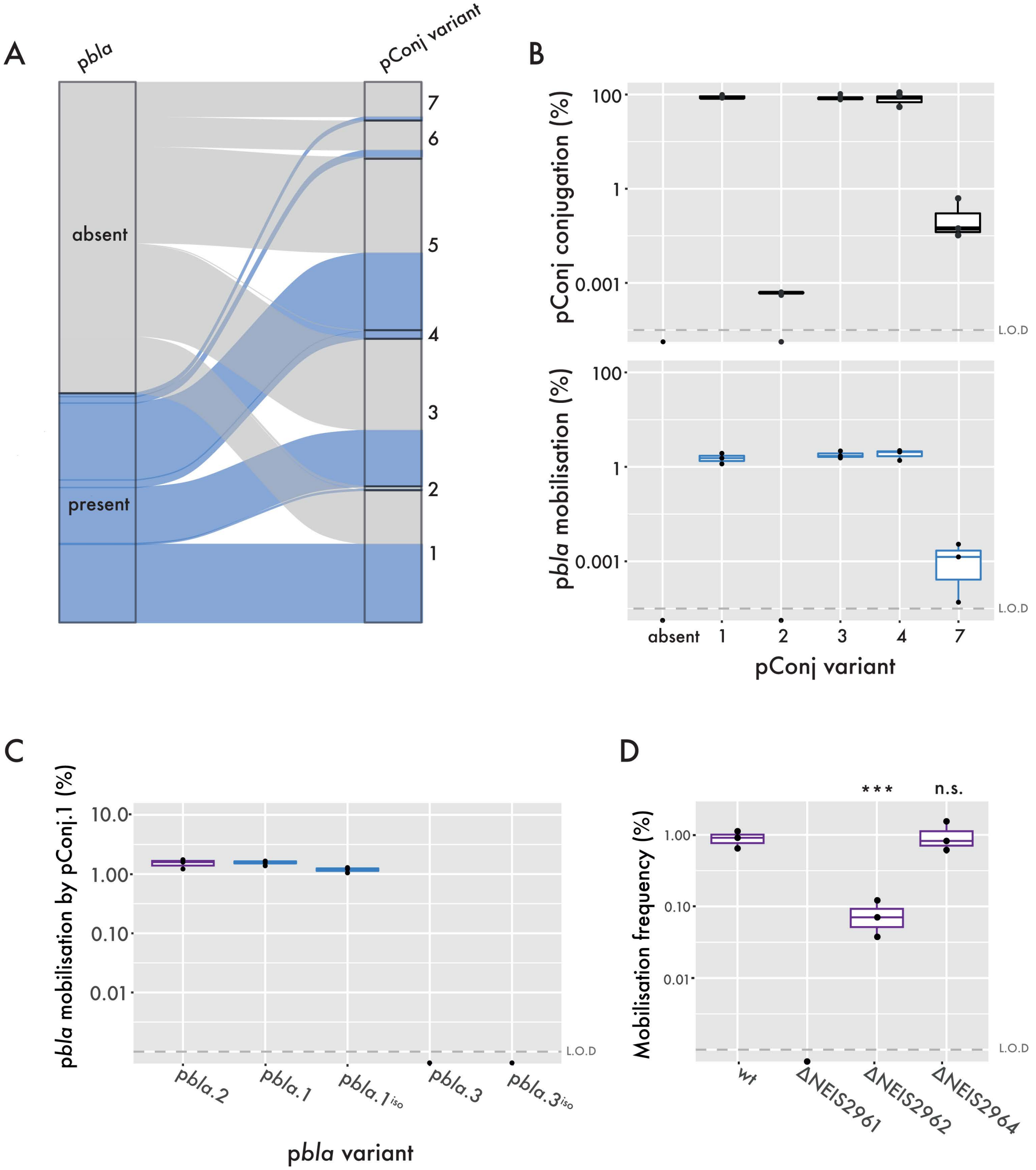

Fig 2. p*bla* mobilisation by pConj. (A) Sankey plot of pConj carrying isolates (n = 4,883 isolates) [5], displaying the presence of p*bla* (left) and co-occurrence of p*bla* with individual pConj variants (right). (B) Conjugation rates of pConj variants (top) and the mobilisation rates of co-located p*bla*.1 (bottom). The limit of detection (L.O.D.) is indicated as a dashed line. (C) Mobilisation rates of wild type and isogenic p*bla* variants (p*bla*^{iso}) by pConj.1. (D) The impact of single *mob* gene knockouts in p*bla*.2 on p*bla* mobilisation frequencies. All assays consist of three individual repeats and were analysed by one-way ANOVA with Tukey multiple comparisons; n.s. $p > 0.05$, *** $p < 0.001$.

https://doi.org/10.1371/journal.ppat.1013151.g002

deletion spans *mobA* (NEIS2961, encoding the relaxase [36]), *mobC* (NEIS2962) and NEIS2964. Deletion of *mobA* abolished p*bla* transfer (Fig 2D), indicating that p*bla* mobilisation depends on its own relaxase and not the pConj relaxase. Removal of NEIS2962 significantly reduced p*bla* mobilisation (p = 0.01, Fig 2D). NEIS2962 is related to MobC from *E.*

coli plasmid RSF1010, which unwinds DNA at the *oriT* [37]. NEIS2962 homodimers are structurally related to MobC and predicted to recognise the p*bla oriT* but not a scrambled *oriT* sequence (S4 Fig). Deletion of both copies of NEIS2964 did not impact p*bla*.2 transfer.

TEM-135 confers increased penicillin resistance

If a plasmid is beneficial to its host, one would expect that plasmid carrying isolates to be highly prevalent in a lineage, due to their competitive advantage of isolates without the plasmid. Therefore, to assess the prevalence of p*bla* in certain lineages, we resolved the gonococcal population structure by clustering isolates according to allelic differences in loci that are core to the gonococcus [38], and defined *N. gonorrhoeae* core genome clusters (Ng_cgc_{400}) with a cut-off of 400 allelic differences. Although p*bla*.3 is immobile, it is highly prevalent in Ng_cgc_{400}s 25 and 298 with 56.8 and 39.6% of isolates in these lineages carrying p*bla*, respectively, Table 1 and S3 Fig) [5]. This suggests p*bla*.3, which carries TEM-135, confers a benefit to the gonococcus that has led to the clonal expansion of isolates containing this p*bla* variant. To test this, we measured the penicillin MICs conferred by p*bla* variants. Whilst TEM-1 carrying p*bla*.1 and 2 conferred MICs of 8 μg/ml, p*bla*.3 with TEM-135 conferred a significantly higher MIC (32 μg/ml, $p = 0.003$, Fig 3A). To establish whether the TEM variant determines resistance levels, we changed p*bla*.3 TEM-135 into TEM-1 by introducing a T182M substitution. This substitution reduced the MIC conferred by p*bla*.3 to levels of p*bla*.1 and p*bla*.2, demonstrating that TEM-135 confers elevated MICs (Fig 3B). We also compared resistance conferred by TEM-1, $TEM\text{-}1_{P14S}$ and TEM-135 (which together account for >95% of gonococcal TEMs [5]) expressed by p*bla*.2. Again, TEM-135 significantly increased MICs (128 μg/ml *vs.* 8 μg/ml with TEM-1, $p < 0.001$, Fig 3C).

To explore the basis for the different MICs, we assessed cellular TEM levels. Levels of mature TEM-135 (29 kDa) were significantly higher than TEM-1 or $TEM\text{-}1_{P14S}$ (Figs 3D and S5), consistent with increased stability of TEM-135 [14]. In conclusion, the appearance of TEM-135, particularly associated with p*bla*.3, provides a significant benefit to the gonococcus by enhancing resistance against beta-lactams, with MICs correlating with cellular TEM levels.

Successful p*bla* variants have evolved with reduced fitness costs

Plasmids often impose fitness costs, disadvantaging isolates that carry plasmids [39]. We therefore assessed the fitness costs of p*bla* by introducing p*bla*.1 into isolates from a range of lineages (S3 Fig) and competing plasmid-carrying *vs.* plasmid-free strains over 24 hrs. p*bla*.1 had no detectable fitness cost in any isolate (Fig 4A), consistent with its continued prevalence in the gonococcus (Fig 4B). We also compared the fitness costs of the three p*bla* variants in wild-type FA1090. In contrast to p*bla*.1 and p*bla*.3 which impose no fitness cost, p*bla*.2 inflicts a significant fitness cost which was evident

Table 1. The percentage of isolates carrying p*bla* in three largest Ng_cgc400 that carry each variant.

Ng_cgc_{400}	Number of isolates	p*bla* variant(s)	p*bla* carriage (%)
21	574	1	64.3
33	476	1	51.9
187	94	1	62.8
3	4545	1/ 2/ 3	1.2/ 0.5/ 0.3
29	473	1/ 2	14/ 18
175	318	2	8.5
25	346	3	56.8
298	101	3	39.6
391	31	3	87.1

https://doi.org/10.1371/journal.ppat.1013151.t001

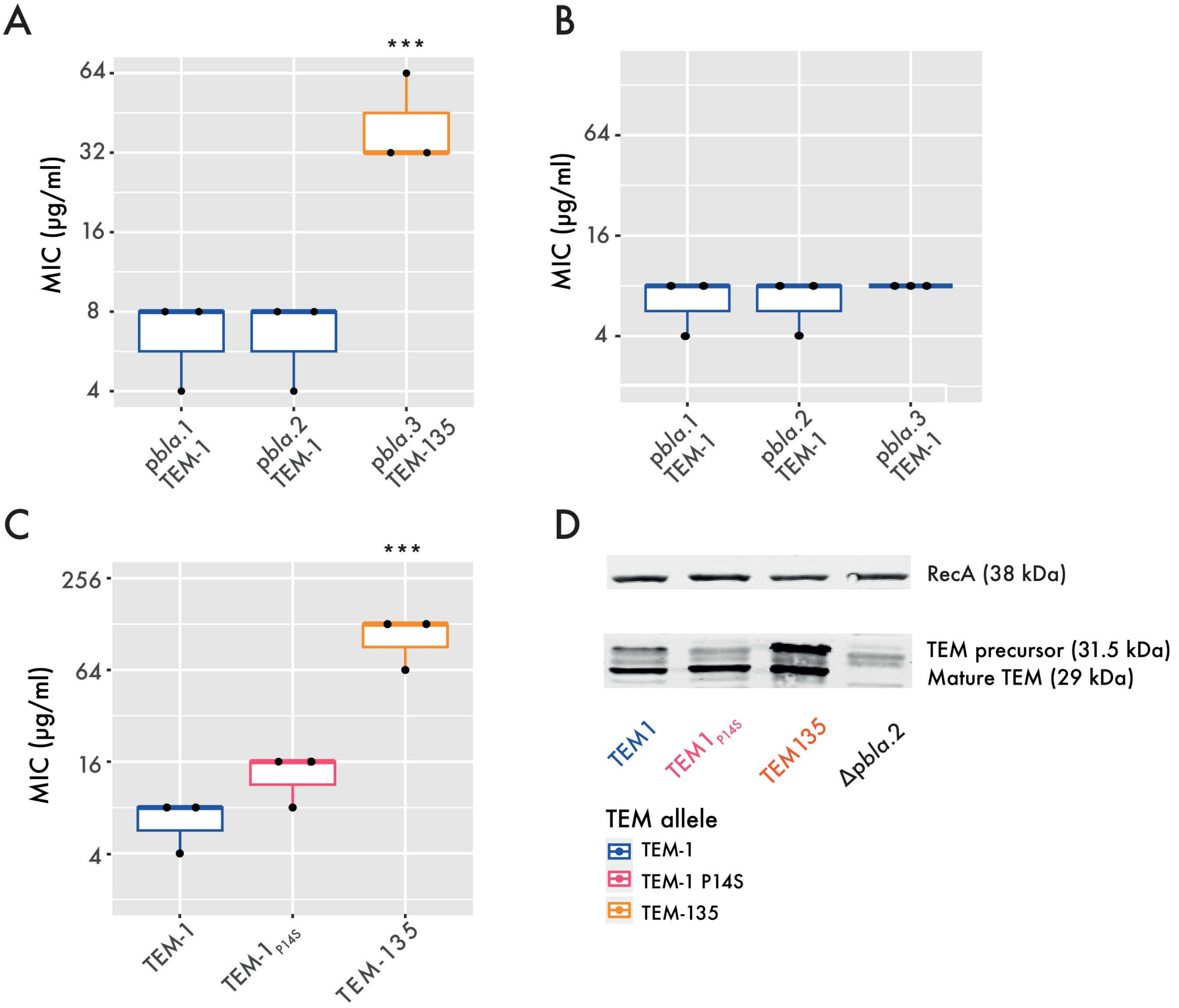

Fig 3. TEM-135 increases the MIC. (A) Penicillin G MICs of p*bla* variants in FA1090 isogenic strain background (one-way ANOVA on log_2-transformed MIC values with Tukey multiple comparisons of means; *** $p < 0.001$). (B) MICs of TEM-1 in different p*bla* variant backbones. (C) MICs of different TEM variants in p*bla*.2 backbone (one-way ANOVA on log_2-transformed MIC values; *** $p < 0.001$). (D) Cellular levels of TEM variants were assessed by Western blot analysis. The image is representative of three biological repeats.

https://doi.org/10.1371/journal.ppat.1013151.g003

within 24 hrs (Fig 4C). This is associated with a higher copy number for p*bla*.2 (>6 copies/chromosome) than the other variants (1–2 copies/chromosome, Fig 4D).

The fitness costs of p*bla*.2 could explain its decreasing prevalence seen when analysing all available WGS in the PubMLST database from 2010 onwards (Fig 4B). To account for any bias in sampling, we also examined the prevalence of p*bla*.1 and p*bla*.2 within a lineage (Ng_cgc_{400} 29) which harbours both these variants. Between 2010 and 2020, there has been a shift from p*bla*.2 to p*bla*.1 in this lineage. In 2011, 55.6% of p*bla* sequences were p*bla*.2 and 33.3% p*bla*.1, while only p*bla*.1 was recovered from isolates of Ng_cgc_{400} 29 after 2018 (Fig 4E).

As the observed changes in p*bla* variant prevalence could result from uneven sampling, we further assessed the relative success of p*bla* variants by their abundance within lineages; if a strain acquires a plasmid which offers an advantage

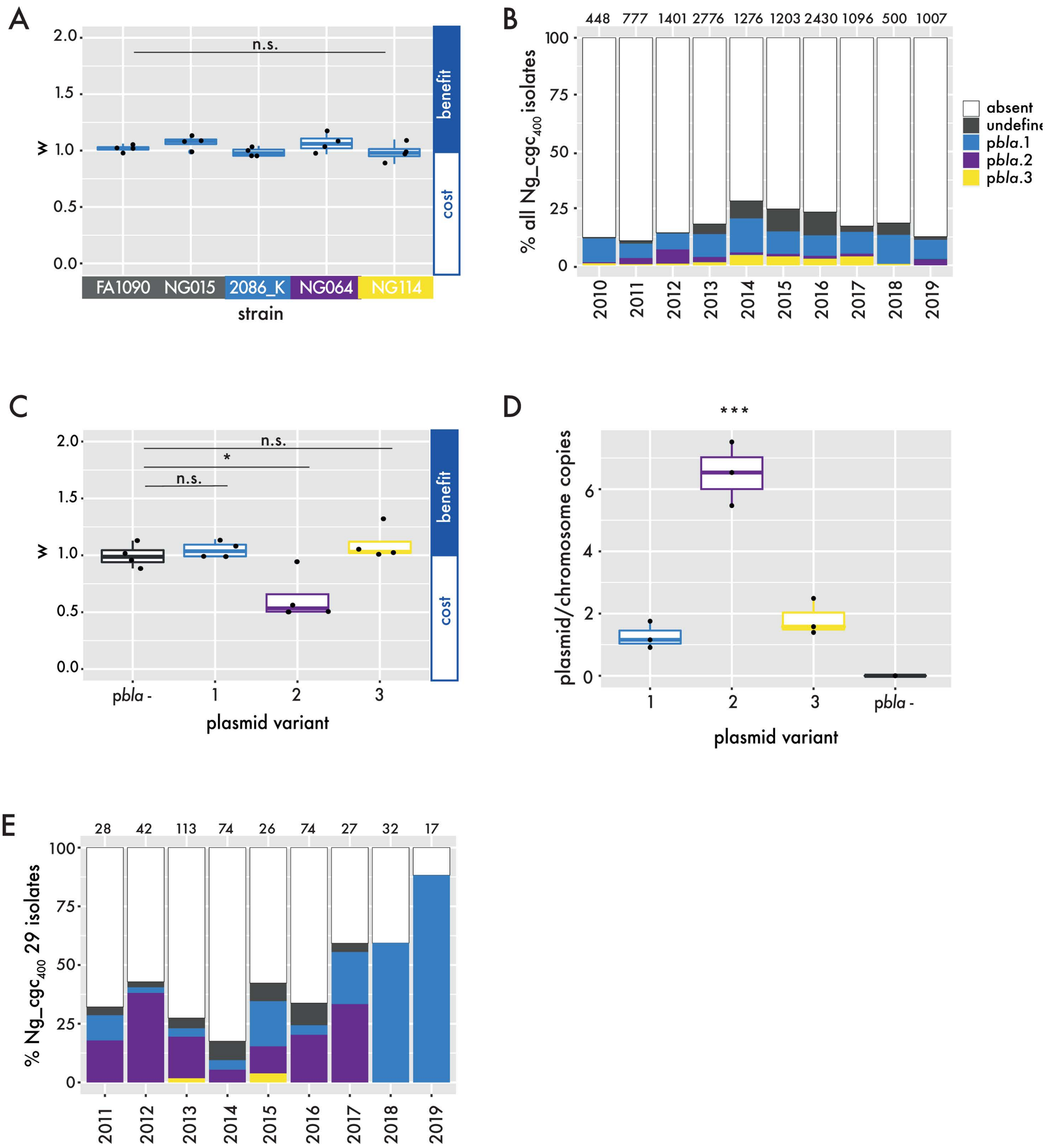

Fig 4. Impact of p*bla*-imposed fitness cost on its prevalence in the population. (A) Fitness cost (w) of p*bla*.1 in clinical isolates from different p*bla*-free (grey) or p*bla*-associated lineages (light blue, p*bla*.1-associated; purple, p*bla*.1/p*bla*.2-associated; yellow, p*bla*.3-associated). $w > 1$ indicates a benefit, whereas $w < 1$ signifies a cost of plasmid carriage. (B) Proportional p*bla* carriage in gonococcal isolates deposited on PubMLST between 2010

and 2019 (n = 12,914 isolates). Colours show p*bla* variant carried and numbers above bars indicate the number of samples in the respective year. (C) Fitness cost of p*bla* variants in FA1090 isogenic strain background were assessed in four independent replicates (one-way ANOVA with Tukey multiple comparisons, n.s. $p > 0.05$; * $p < 0.05$). (D) Copy number of p*bla* in FA1090 isogenic strain background was assessed by ddPCR (one-way ANOVA with Tukey multiple comparisons; *** $p < 0.001$). (E) p*bla* carriage in isolates from the p*bla*.1/p*bla*.2-associated Ng_cgc_{400} 29 between 2011 and 2019 (n = 433 isolates). Bar colours indicate p*bla* variant and numbers above the bars specify the number of samples in the respective year.

https://doi.org/10.1371/journal.ppat.1013151.g004

it will undergo clonal expansion and outcompete other strains belonging to the same lineage but lacking the plasmid. Therefore, we examined the percentage of strains with p*bla* in the three largest lineages carrying each p*bla* variant. p*bla*.1 is highly prevalent in lineages (50–60% of p*bla* carriage in major p*bla*.1 lineages, Table 1). In contrast, p*bla*.2 is only present at low frequency in lineages (<20%, Table 1), or is found in lineages with other variants. p*bla*.3 which expresses TEM-135 with no obvious fitness cost is found in a high percentage of strains in a lineage (39–84%).

Taken together, fitness costs imposed by p*bla*.2 are consistent with its low prevalence in the gonococcal population compared with p*bla*.1. p*bla*.3 with TEM-135, which arose from p*bla*.2, confers elevated penicillin resistance without fitness costs, and is associated with the success of a small group of related lineages.

Discussion

Plasmids are important vehicles for AMR, with resistance plasmids amongst the most diverse and mobile [40]. Here, we investigated the origin of the relatively conserved beta-lactamase plasmid p*bla* which is largely found in the gonococcus, a WHO priority pathogen. Our analysis indicates that p*bla* was likely acquired by *N. gonorrhoeae* from another cause of STI, *H. ducreyi*. We describe the evolution of p*bla* since its emergence in *N. gonorrhoeae*, and its association with pConj. Gene loss and the appearance of novel TEM alleles influence the benefits and costs of p*bla* to the gonococcus. These traits are associated with the success and distribution of p*bla* variants within the gonococcal population.

In women, *N. gonorrhoeae* primarily causes cervicitis, while *H. ducreyi* causes ulcers at the vaginal entrance and cervix. In men, *N. gonorrhoeae* primarily causes urethritis and *H. ducreyi* mainly causes penile ulcers [41]. Therefore, these species can occupy the same niche, providing ample opportunities for gene transfer. The presence of p*bla*.1- and p*bla*.2-like plasmids in *H. ducreyi* could indicate independent introductions of p*bla*.1 and p*bla*.2 into the gonococcus or independent emergence of p*bla*.1 in *H. ducreyi* and *N. gonorrhoeae*. Whilst we cannot reject either scenario, the independent emergence of p*bla*.1 in *H. ducreyi* through the deletion of *repB*/NEIS2964 allele 2 and *repB*/NEIS2964 allele 3, respectively, is the most parsimonious explanation.

p*bla* is found relatively frequently in *N. gonorrhoeae* and *H. ducreyi*, which inhabit the genitourinary tract, but is seldom present in pathogens which inhabit the nasopharynx (*e.g., H. influenzae* and *N. meningitidis*); p*bla* was not detected in any non-invasive *Neisseria* spp. (41,158 isolates, 30 species). This could reflect the renal excretion of beta-lactams [42], favouring p*bla* carriage among bacteria inhabiting the urogenital tract compared with other sites. The meningococcal urethritis clade (NmUC) evolved from ST-11 *N. meningitidis* by acquiring genetic elements from *N. gonorrhoeae* [43,44]. So far, p*bla* has not been reported in NmUC. However, we previously found an ST-11 *N. meningitidis* isolate harbouring p*bla* [5]. This raises the possibility that NmUC might acquire p*bla*, which could provide an entry point of this plasmid into the meningococcal population, and the emergence of beta-lactamase producing *N. meningitidis*.

Plasmids can be successful in bacterial populations either by horizontal transmission and their ability to spread into diverse lineages, or through vertical transmission through their stable inheritance while being beneficial to their host. Our data indicates that the success of p*bla* depends both on its own mobility and its association with pConj variants that can effectively mediate its spread. Given the higher rates of pConj conjugation (>75%) compared with p*bla* mobilisation (~1%), the spread of p*bla* into a lineage is likely to be accompanied by pConj, maintaining the close association between these plasmids. Interesting, unlike many other conjugative plasmids [45], pConj lacks entry exclusion, so p*bla* can enter bacteria

already containing pConj, allowing proliferation of bacteria with successful p*bla*/pConj pairs, such as p*bla*.1/pConj.1, across the gonococcal population.

The most frequent and widespread p*bla* variant, p*bla*.1, is mobilised efficiently by common pConj variants and does not impose fitness costs. p*bla*.2 is also mobile but imposes fitness costs. Compared with p*bla*.1, p*bla*.2 has a second replication initiation protein and additional origins of replication (*i.e., ori2* and *ori3*) [5,46] which might increase copy number and/or impose the fitness costs of p*bla*.2; plasmid Rep proteins can sequester host DNA replication machinery [39], causing fitness costs. We found that p*bla*.2 is present in lower prevalence in lineages than p*bla*.1, with evidence of a shift from p*bla*.2 to p*bla*.1 in a single lineage (Ng_cgc$_{400}$ 29) over time. Whilst the observed shift in p*bla* variants could reflect sampling bias, it is consistent with strains carrying p*bla*.2 being outcompeted by plasmid-free isolates or those with other p*bla* variants. Further evidence is provided by a longitudinal 10 year study of over 1,700 gonococcal isolates from a single city in China (Guangzhou), which reported a marked increase in p*bla*.1 (p*bla* Africa, from 18.4 to > 90% of isolates) with a concurrent fall in p*bla*.2-containing strains (from 81.6 to 7.6%) due to expansion of successful clones harbouring p*bla*.1 [47].

p*bla*.3-associated lineages have undergone clonal expansion indicating its successful adaptation to the gonococcus. Phylogenetic analysis indicates that TEM-135 originally arose in p*bla*.2. However, despite increased resistance levels conferred by TEM-135, the fitness cost of p*bla*.2 has likely undermined the success of TEM-135 in this p*bla* variant. We found that p*bla*.3 evolved from TEM-135 carrying p*bla*.2 through gene loss. This prevented the plasmid from being mobile, but with the trade-off of avoiding fitness costs. Consequently, the prevalence of p*bla*.3 in gonococci is not due to transfer between isolates, but through the expansion of p*bla*.3-carrying isolates, potentially through the increased beta-lactam resistance associated with TEM-135 and/or its association with an otherwise successful lineage. Further work is required to distinguish between these and other possibilities.

Since the emergence of gonococcal p*bla* in the 1970s, the evolutionary trajectory of this plasmid has been marked by its association with pConj variants that enable its spread through the population, the appearance of plasmid variants with minimal costs, and emergence of TEMs promoting higher resistance (*e.g.,* TEM-135). A major concern is that the ESBL-permissive M182T substitution in TEM-135 is already widespread in gonococci [5,6], especially in p*bla*.3.

The intimate relationship between p*bla* and pConj also highlights the threat posed by increased use of tetracyclines, as already witnessed in LMICs where gonococci have remarkably high plasmid carriage [4,48]. Similarly, the implementation of Doxy-PEP will increase selection for the carriage of pConj in gonococci and will thence select the isolates/lineages harbouring the plasmid. A significant threat is posed by isolates carrying pConj as well as chromosomal mutations conferring cefotaxime resistance [49]. The spread of these isolates, as well as p*bla* (which itself could become an ESBL-plasmid) have the potential to undermine the successful treatment of cases and their contacts with third-generation cephalosporins, the first-line antibiotics currently used to control gonococcal infection.

Methods

Analysis of *Haemophilus* spp. and *Neisseria* spp. genomes

Tn*2*-carrying isolates of *Haemophilus* spp. and *Neisseria* spp. were identified querying Tn*2* (GenBank accession: LC091537.1) against *Haemophilus* (accessed 8/4/2025, 6,403 isolates, 12 species) and *Neisseria* (accessed 24/07/2024, 41,158 isolates, 33 species) sequences in PubMLST [21] (blastN word size: 11, scoring: reward: 2; penalty: -3; gap open: 5; gap extend: 2, sequence identity >99%, alignment length >50% query). p*bla*-like plasmids were confirmed by the presence of NEIS2960 (sequence identity >80%; alignment length >50%) and NEIS2358, and NEIS2961[5].

Plasmid variants

p*bla* and pConj were analysed in 15,529 gonococcal WGS on PubMLST (accessed 28/07/2022, S4 Table) [5,21] with isolates from 1928-2022 and 66 countries. NEIS2220 indicates the presence of pConj, and variants were defined according

to gene presence/absence and specific alleles of plasmid genes [6]. p*bla* variants were typed using the Ng_p*bla*ST scheme [5]. For the population structure, isolates were grouped into core genome clusters according to the allelic profile of 1,668 core genes [38]; isolates were grouped with a cut-off of 400 allelic differences (Ng_cgc_{400}).

Phylogenetic analyses

A subset of 414 p*bla*-carrying isolates conserving the ratio of p*bla* variants (70% p*bla*.1, 14% p*bla*.2, 16% p*bla*.3, S3 Table) [5] was selected to investigate the phylogenetic relationship of p*bla* variants. This included all p*bla*-containing isolates pre-dating 2000 (n = 35). Isolates between 2000 and 2022 (n = 379) were randomly selected using the r sample function [5]. Snippy v4.6.0 mapped plasmid reads to *H. ducreyi* DMC64 p*bla* (minimum coverage, 4 and base quality, 25). Multiple sequence alignments were generated with snippy-core v4.6.0/snippy-clean v4.6.0. Maximum likelihood trees were generated using RaxML-ng v1.2.2 [50] with 100 bootstrap replicates, rooted at *H. ducreyi* DMC64 p*bla*, and visualised with ape [51] and ggtree [52,53].

Structure predictions

Analysis of NEIS2962 and RSF1010 MobC (GenBank accession: S96966.1) homodimers and NEIS2962 with p*bla oriT* [36] were performed using AlphaFold 3 [54] and PyMol v2.5.4 [55]. Charge distributions were visualised with the Adaptive Poisson-Boltzmann Solver (APBS) electrostatics tool [56].

Bacterial strains/growth

Strains and plasmids used in this study are listed in S5 and S6 Tables, respectively. *E. coli* DH5α was grown on Luria-Bertani (LB) agar or in liquid LB shaking at 180 rpm. *N. gonorrhoeae* was grown on Gonococcal Base Media (GCB) agar plates or liquid media (GCBL) [57] supplemented with 1% Vitox (Oxoid) at 37°C in 5% CO_2. *H. ducreyi* was grown on chocolate agar plates supplemented with 1% IsoVitaleX at 35°C in 5% CO_2. Antibiotics were added as follows: for *E. coli*, carbenicillin 100 μg/ml; for *N. gonorrhoeae*, carbenicillin 2.5 μg/ml; erythromycin 1 μg/ml; kanamycin 50 μg/ml, and tetracycline 2 μg/ml.

Characterisation of *H. ducreyi* plasmids

Genomic DNA was isolated from *H. ducreyi* by harvesting bacteria from plates and the DNeasy Blood/Tissue Kit (Qiagen) with the modifications that cells were incubated in lysis buffer with 20 mg/ml lysozyme at 37°C for 2 hours and then proteinase K overnight at 56°C. Plasmids were analysed by Sanger sequencing using primers listed in S7 Table. Sequence similarity of plasmid sequences was assessed with ClustalW with default parameters [58] and sequences were mapped onto *H. ducreyi* DMC64 p*bla* using Snapgene v6.1.1 (Insightful Science; available from snapgene.com) to investigate deletion sites.

Transformation of gonococci

For electroporation, bacteria grown on GCB agar were resuspended in PBS (Sigma), adjusted to $5x10^7$ CFU/ml then washed three times with 20% glycerol/ 1% MOPS (Sigma); electroporation was performed with 2.5 kV, 200 Ω, 25 mF. Cells were recovered in 1 ml of GCBL with 2% Vitox and plated on GCB agar. Plates were incubated for 3 hours, cells collected, and then transferred to selective media.

Δ*pilD*::*ermC* and Δ*pilD*::*aph* constructs were transformed into *N. gonorrhoeae* as described previously [27,29]. In brief, 1 μg of DNA was spotted onto plates, allowed to dry, and bacteria streaked over the spots. Plates were incubated for 8 hours, then bacteria were transferred onto selective agar. Transformants confirmed by PCR/Sanger sequencing.

Plasmid modifications

All primers are listed in S8 Table. To generate p*bla*.1^{iso} and p*bla*.3^{iso}, p*bla*.2 was cut with *Hin*dIII-HF and *Pvu*II-HF (NEB), respectively. p*bla*.1^{iso} was amplified with primers TE18/19 and PrimeSTAR GXL polymerase (Takara Bio); Gibson assembly was performed with primers TE20/21. p*bla*.3^{iso} was amplified in two fragments with TE7/TE17 and TE9/TE16. Plasmids were assembled using Gibson Hifi (NEB) and transformed into *E. coli* DH5α.

Point mutations in *bla*TEM were introduced using the RAIR method [59]. PCRs with primers (TE56/TE57, p*bla*.2 TEM-135; TE63/TE64, p*bla*.2$^{TEM\text{-}1\ P14S}$; TE65/TE66, p*bla*.3$^{TEM\text{-}1}$) were performed using Herculase II polymerase (Agilent), with p*bla*.2 or p*bla*.3 as template. Products were purified (Promega Wizard PCR Clean-up) and transformed into *E. coli* DH5α.

tetM$^+$ pConj.7 was constructed by amplifying *tetM* from *N. gonorrhoeae* WHO N using primers TE34/TE35. Flanking regions were amplified with primers TE36/37 and TE38/39, then joined by Gibson assembly; the product was amplified with TE36/39, then introduced into *N. gonorrhoeae* NG028 by transformation. All constructs were confirmed by sequencing.

Conjugation and mobilisation assays

Donor (Δ*pilD*::*ermC*) and recipient (Δ*pilD*::*aph*) strains grown overnight were inoculated in 5 ml GCBL/1% Vitox at an OD_{600} of 0.1 and grown to mid-exponential phase (OD_{600} 0.6 - 0.8). The bacterial density was adjusted to 10^8 CFU/ml and donor and recipient strains mixed in a 10:1 ratio. Bacteria (5 µl) were spotted onto GCB agar and incubated for 6 hours at 37°C, 5% CO_2, harvested in 200 µl GCBL, then plated to GCB agar with antibiotics. Conjugation and mobilisation frequencies were defined as the number of transconjugants/recipients (n = 3). Plasmids used in the different assays are listed in S6 Table together with the WGS of the corresponding isolates available on PubMLST.

Competition assays to determine fitness costs

Plasmids were introduced into FA1090 Δ*pilD*::*ermC* and competed against FA1090 Δ*pilD*::*aph* (n = 4). Bacteria in PBS were adjusted to an OD_{600} 1, mixed 1:1, diluted to 10^5 CFU/ml and added to 200 µl Fastidious Broth [60] then grown at 37°C, 5% CO_2, shaking at 180 rpm. After 24 hours, strains were enumerated by spotting on selective media. Fitness costs (w) were calculated by:

$$w = \frac{ln(N_f/N_i)}{ln(N_{f,pbla}/N_{i,pbla})}$$

(w, relative fitness of p*bla*$^+$ *vs*. p*bla*$^-$ strains; N_i and N_f, p*bla*$^-$ strain at the beginning/end, respectively; $N_{i,pbla}$ and $N_{f,pbla}$, same for p*bla*$^+$ strain).

Antibiotic susceptibility testing

Penicillin G MICs were assessed using the broth microdilution method [61] in 96-well plates with 2-fold Penicillin dilutions in water (50 µl); strains grown overnight on GCB agar were resuspended in PBS (Sigma), then diluted in 2x FB/2% Vitox to 10^5 CFU/ml. Bacteria (50 µl) were transferred into each well and incubated for 24 hours.

SDS page and Western blot analysis

Bacteria were grown to mid-exponential phase, added to an equal volume of 2x SDS-PAGE buffer, run on 12% SDS-polyacrylamide gels, and transferred to Protan nitrocellulose membranes (GE Healthcare) using the Trans-Blot Turbo System (Bio-Rad). Membranes were blocked in PBS/0.5% Tween-20/5% milk, washed thrice and incubated with the primary antibodies (Rabbit anti-RecA, Abcam, ab63797, 1:5,000; Mouse anti-TEM, Abcam, 8A5.A10, 1:1,000) for 2 hours. After

washing, membranes were incubated with secondary antibodies (LI-COR Biosciences, 925–68071 IRDye 680RD Goat anti-Rabbit IgG and 925–32210 IRDye 800CW Goat anti-Mouse IgG) at a final dilution of 1:10,000 for 1 hour, washed, then imaged using LI-COR Biosciences.

Plasmid copy number

Copy number of *recA* and plasmid *tnpR* were quantified using the QX200 Droplet Digital PCR system (ddPCR, Bio-Rad) as described previously [62]. ddPCR contained 1x EvaGreen super mix (Bio-Rad), and TE79/TE80 (*recA*, S8 Table) or TE81/TE82 (*tnpR*, S8 Table). After thermal cycling, data were analysed using the QX200 Droplet Reader with QuantaSoft software (Bio-Rad).

TEM mRNA expression

p*bla*.1^{TEM-1} carrying isolates were sub-cultured to mid-exponential phase in 5 ml GCBL/1% Vitox. Cells were harvested from 1 ml of culture and RNA was extracted using the Qiagen RNeasy Mini Kit together with the RNA protect Reagent (Qiagen, #76506) and on column RNase-free DNase I (Qiagen, #79254) treatment according to the manufacturer's instruction (protocol RY28). RNA was subsequently reverse transcribed to cDNA using the LunaScript RT SuperMix Kit (NEB, #E3010). The cDNA was used in ddPCRs with primers TE79/80 (*recA*) and TE97/98 (*bla*TEM, S8 Table). The no-RT and no template reactions served as negative controls.

Statistics and data analysis

Data analysis was performed in R version 4.1.1 using base R and the tidyverse package [63]. Plots were generated with ggplot2 [64]. A p value <0.05 was considered statistically significant.

Supporting information

S1 Fig. (A) Analysis of the Tn*2* deletion region: the truncation affecting *tnpR* in gonococcal p*bla*.1 and p*bla*.2 differ by a single nucleotide. Schematic representation of *tnpR* sequences of gonococcal and *H. ducreyi* p*bla*s is shown on the bottom with the SNP displayed as a white line and the site of the Tn*2* truncation in relation to *H. ducreyi* p*bla* indicated with a triangle. Displayed above is the nucleotide alignment of gonococcal p*bla* to *H. ducreyi* DMC64 p*bla*.2. (B) Sequence alignment of NEIS2964 alleles -1 to -3) with SNPs highlighted in white.
(AI)

S2 Fig. p*bla*.1 mobilisation by pConj.1 in isogenic matings. (A) p*bla*.1 mobilisation in the *N. gonorrhoeae* strains FA1090 and 2086_K (Welch two-sample t-test, p = 0.66). (B) Mobilisation rates of p*bla*.1 into FA1090 with or without pConj (Welch two-sample t-test, p = 0.87).
(AI)

S3 Fig. Minimum spanning trees of *N. gonorrhoeae* clustered by core genome allelic differences using Grapetree [65] with distribution of p*bla* variants. Individual dots represent isolates that are coloured according to Ng_cgc_{400} (A) or p*bla* variant carried (B). Strains used in this study are indicated in panel A.
(AI)

S4 Fig. (A) Alignment of MobC from *E. coli* plasmid RSF1010 (Genbank accession: S96966.1) and NEIS2962 from p*bla*.2 (Genbank accession: NZ_LT591911). Amino acid sequences were aligned with COBALT [66] and the alignment visualised with Esprit [67]. Identical residues are shown in white on red background, residues with a similarity score >0.7 are framed in blue and the remaining residues are shown in black. (B) Superimposed AlphaFold structure prediction of MobC from the *E. coli* plasmid RSF1010 (salmon, Genbank accession: S96966.1) and NEIS2962 (blue) dimers (Match

Align: 677.7, RMSD: 0.775Å) (C) Electrostatics prediction of NEIS2962 homodimer with *oriT* sequence using the Adaptive Poisson Boltzman Solver Electrostatics Plugin. Negatively and positively charged regions are shown in red and blue, respectively.
(AI)

S5 Fig. Cellular levels of different TEM variants in an isogenic FA1090 background. TEM/RecA ratios of whole cell lysates were visualised by Western blot analysis and quantified with the LI-COR system (one-way ANOVA with Tukey multiple comparisons, n.s. $p > 0.05$; *** $p < 0.001$).
(AI)

S1 Table. Genes in *Haemophilus* spp. related to p*bla.*
(XLSX)

S2 Table. Genes in *H. ducreyi* isolates related to p*bla.*
(XLSX)

S3 Table. Examples of gonococcal p*bla* used for phylogeny.
(XLSX)

S4 Table. *N. gonorrhoeae* isolates used for phylogeny.
(XLSX)

S5 Table. Bacterial strains used in this study.
(XLSX)

S6 Table. Plasmids used in this study.
(XLSX)

S7 Table. Primers used for sequencing.
(XLSX)

S8 Table. Primers used for strain construction.
(XLSX)

Author contributions

Conceptualization: Tabea A Elsener, Ana Cehovin, Stanley M. Spinola, Martin C.J. Maiden, Christoph M. Tang.

Data curation: Tabea A Elsener, Ana Cehovin, Stanley M. Spinola.

Formal analysis: Tabea A Elsener, Ana Cehovin, Kate Fortney, Martin C.J. Maiden, Christoph M. Tang.

Funding acquisition: Christoph M. Tang.

Investigation: Tabea A Elsener, Connor Philp, Kate Fortney, Stanley M. Spinola.

Methodology: Tabea A Elsener.

Project administration: Christoph M. Tang.

Resources: Kate Fortney, Stanley M. Spinola, Martin C.J. Maiden.

Software: Tabea A Elsener, Martin C.J. Maiden.

Supervision: Tabea A Elsener, Ana Cehovin, Martin C.J. Maiden, Christoph M. Tang.

Validation: Tabea A Elsener, Kate Fortney, Christoph M. Tang.

Visualization: Tabea A Elsener, Ana Cehovin.

Writing – original draft: Tabea A Elsener, Ana Cehovin, Connor Philp, Kate Fortney, Stanley M. Spinola, Martin C.J. Maiden, Christoph M. Tang.

Writing – review & editing: Tabea A Elsener, Ana Cehovin, Kate Fortney, Stanley M. Spinola, Martin C.J. Maiden, Christoph M. Tang.

References

1. World Health Organization. Multi-drug resistant gonorrhoea. 2021 Available from: https://www.who.int/news-room/fact-sheets/detail/multi-drug-resistant-gonorrhoea
2. Aitolo GL, Adeyemi OS, Afolabi BL, Owolabi AO. *Neisseria gonorrhoeae* Antimicrobial Resistance: Past to Present to Future. Curr Microbiol. 2021;78(3):867–78. https://doi.org/10.1007/s00284-021-02353-8 PMID: 33528603
3. Stewart J, Oware K, Donnell D, Violette LR, Odoyo J, Soge OO, et al. Doxycycline Prophylaxis to Prevent Sexually Transmitted Infections in Women. N Engl J Med. 2023;389(25):2331–40. https://doi.org/10.1056/NEJMoa2304007 PMID: 38118022
4. Cehovin A, Harrison OB, Lewis SB, Ward PN, Ngetsa C, Graham SM, et al. Identification of Novel *Neisseria gonorrhoeae* Lineages Harboring Resistance Plasmids in Coastal Kenya. J Infect Dis. 2018;218(5):801–8. https://doi.org/10.1093/infdis/jiy240 PMID: 29701830
5. Elsener TA, Jolley KA, Sanders E, Maiden MCJ, Cehovin A, Tang CM. There are three major Neisseria gonorrhoeae beta-lactamase plasmid variants which are associated with specific lineages and carry distinct TEM alleles. Microb Genom. 2023;9(7).
6. Cehovin A, Jolley KA, Maiden MCJ, Harrison OB, Tang CM. Association of *Neisseria gonorrhoeae* Plasmids With Distinct Lineages and The Economic Status of Their Country of Origin. J Infect Dis. 2020;222(11):1826–36. https://doi.org/10.1093/infdis/jiaa003 PMID: 32163577
7. Dönhöfer A, Franckenberg S, Wickles S, Berninghausen O, Beckmann R, Wilson DN. Structural basis for TetM-mediated tetracycline resistance. Proc Natl Acad Sci U S A. 2012;109(42):16900–5.
8. Ashford WA, Golash RG, Hemming VG. Penicillinase-producing *Neisseria gonorrhoeae*. Lancet. 1976;2(7987):657–8. https://doi.org/10.1016/s0140-6736(76)92467-3 PMID: 60519
9. Phillips I. Beta-lactamase-producing, penicillin-resistant gonococcus. Lancet. 1976;2(7987):656–7. https://doi.org/10.1016/s0140-6736(76)92466-1 PMID: 60518
10. Salverda MLM, De Visser JAGM, Barlow M. Natural evolution of TEM-1 β-lactamase: experimental reconstruction and clinical relevance. FEMS Microbiol Rev. 2010;34(6):1015–36. https://doi.org/10.1111/j.1574-6976.2010.00222.x PMID: 20412308
11. Hazra A, Collison MW, Davis AM. CDC Sexually Transmitted Infections Treatment Guidelines, 2021. JAMA. 2022;327(9):870–1. https://doi.org/10.1001/jama.2022.1246 PMID: 35230409
12. Biswas GD, Blackman EY, Sparling PF. High-frequency conjugal transfer of a gonococcal penicillinase plasmid. J Bacteriol. 1980;143(3):1318–24. https://doi.org/10.1128/jb.143.3.1318-1324.1980 PMID: 6773927
13. Pagotto F, Aman AT, Ng LK, Yeung KH, Brett M, Dillon JA. Sequence analysis of the family of penicillinase-producing plasmids of *Neisseria gonorrhoeae*. Plasmid. 2000;43(1):24–34. https://doi.org/10.1006/plas.1999.1431 PMID: 10610817
14. Huang W, Palzkill T. A natural polymorphism in beta-lactamase is a global suppressor. Proc Natl Acad Sci U S A. 1997;94(16):8801–6. https://doi.org/10.1073/pnas.94.16.8801 PMID: 9238058
15. Brunton J, Meier M, Erhman N, Clare D, Almawy R. Origin of small beta-lactamase-specifying plasmids in *Haemophilus* species and *Neisseria gonorrhoeae*. J Bacteriol. 1986;168(1):374–9. https://doi.org/10.1128/jb.168.1.374-379.1986 PMID: 3020002
16. Laufs R, Kaulfers PM, Jahn G, Teschner U. Molecular characterization of a small *Haemophilus influenzae* plasmid specifying beta-lactamase and its relationship to R factors from *Neisseria gonorrhoeae*. J Gen Microbiol. 1979;111(1):223–31. https://doi.org/10.1099/00221287-111-1-223 PMID: 110907
17. Brunton J, Clare D, Meier MA. Molecular epidemiology of antibiotic resistance plasmids of *Haemophilus* species and *Neisseria gonorrhoeae*. Rev Infect Dis. 1986;8(5):713–24. https://doi.org/10.1093/clinids/8.5.713 PMID: 3024290
18. Brunton JL, Maclean I, Ronald AR, Albritton WL. Plasmid-mediated ampicillin resistance in Haemophilus ducreyi. Antimicrob Agents Chemother. 1979;15(2):294–9. https://doi.org/10.1128/AAC.15.2.294 PMID: 311618
19. Laufs R, Kaulfers PM. Molecular characterization of a plasmid specifying ampicillin resistance and its relationship to other R factors from Haemophilus influenzae. J Gen Microbiol. 1977;103(2):277–86. https://doi.org/10.1099/00221287-103-2-277 PMID: 304471
20. Elwell LP, Saunders JR, Richmond MH, Falkow S. Relationships among some R plasmids found in Haemophilus influenzae. J Bacteriol. 1977;131(1):356–62. https://doi.org/10.1128/jb.131.1.356-362.1977 PMID: 301523
21. Jolley KA, Bray JE, Maiden MCJ. Open-access bacterial population genomics: BIGSdb software, the PubMLST.org website and their applications. Wellcome Open Res. 2018;3:124. https://doi.org/10.12688/wellcomeopenres.14826.1 PMID: 30345391
22. Heffron F, Sublett R, Hedges R, Jacob A, Falkow S. Origin of the tem-beta-lactamase gene found on plasmids. J Bacteriol. 1975;122(1):250–6.

23. Partridge SR, Hall RM. Evolution of transposons containing *bla*TEM genes. Antimicrob Agents Chemother. 2005;49(3):1267–8. https://doi.org/10.1128/AAC.49.3.1267-1268.2005 PMID: 15728947
24. Gangaiah D, Webb KM, Humphreys TL, Fortney KR, Toh E, Tai A, et al. *Haemophilus ducreyi* Cutaneous Ulcer Strains Are Nearly Identical to Class I Genital Ulcer Strains. PLoS Negl Trop Dis. 2015;9(7):e0003918. https://doi.org/10.1371/journal.pntd.0003918 PMID: 26147869
25. Gangaiah D, Webb KM, Humphreys TL, Fortney KR, Toh E, Tai A, et al. *Haemophilus ducreyi* Cutaneous Ulcer Strains Are Nearly Identical to Class I Genital Ulcer Strains. PLoS Negl Trop Dis. 2015;9(7):e0003918. https://doi.org/10.1371/journal.pntd.0003918 PMID: 26147869
26. Sánchez-Busó L, Golparian D, Parkhill J, Unemo M, Harris SR. Genetic variation regulates the activation and specificity of Restriction-Modification systems in Neisseria gonorrhoeae. Sci Rep. 2019;9(1):14685. https://doi.org/10.1038/s41598-019-51102-2 PMID: 31605008
27. Jones RA, Yee WX, Mader K, Tang CM, Cehovin A. Markerless gene editing in *Neisseria gonorrhoeae*. Microbiology (Reading). 2022;168(6):10.1099/mic.0.001201. https://doi.org/10.1099/mic.0.001201 PMID: 35763318
28. Freitag NE, Seifert HS, Koomey M. Characterization of the pilF-pilD pilus-assembly locus of Neisseria gonorrhoeae. Mol Microbiol. 1995;16(3):575–86. https://doi.org/10.1111/j.1365-2958.1995.tb02420.x PMID: 7565116
29. Yee W-X, Yasir M, Turner AK, Baker DJ, Cehovin A, Tang CM. Evolution, persistence, and host adaption of a gonococcal AMR plasmid that emerged in the pre-antibiotic era. PLoS Genet. 2023;19(5):e1010743. https://doi.org/10.1371/journal.pgen.1010743 PMID: 37186602
30. Getino M, de la Cruz F. Natural and artificial strategies to control the conjugative transmission of plasmids. Microbiol Spectr. 2018;6(1).
31. Pachulec E, van der Does C. Conjugative plasmids of *Neisseria gonorrhoeae*. PLoS One. 2010;5(4):e9962. https://doi.org/10.1371/journal.pone.0009962 PMID: 20376355
32. Marquez C, Xia M, Borthagaray G, Roberts MC. Conjugal transfer of the 3.05 beta-lactamase plasmid by the 25.2 Mda plasmid in *Neisseria gonorrhoeae*. Sex Transm Dis. 1999;26(3):157–9. https://doi.org/10.1097/00007435-199903000-00006 PMID: 10100773
33. Scharbaai-Vázquez R, Candelas T, Torres-Bauzá LJ. Mobilization of the gonococcal 5.2 kb beta-lactamase plasmid pSJ5.2 into Escherichia coli by cointegration with several gram-conjugative plasmids. Plasmid. 2007;57(2):156–64. https://doi.org/10.1016/j.plasmid.2006.07.006 PMID: 17027960
34. Scharbaai-Vázquez R, González-Caraballo AL, Torres-Bauzá LJ. Four different integrative recombination events involved in the mobilization of the gonococcal 5.2 kb beta-lactamase plasmid pSJ5.2 in *Escherichia coli*. Plasmid. 2008;60(3):200–11. https://doi.org/10.1016/j.plasmid.2008.07.004 PMID: 18778732
35. Ramsay JP, Firth N. Diverse mobilization strategies facilitate transfer of non-conjugative mobile genetic elements. Curr Opin Microbiol. 2017;38:1–9. https://doi.org/10.1016/j.mib.2017.03.003 PMID: 28391142
36. Rodriguez-Bonano NM, Torres-Bauza LJ. Molecular analysis of *oriT* and MobA protein in the 7.4 kb mobilizable beta-lactamase plasmid pSJ7.4 from *Neisseria gonorrhoeae*. Plasmid. 2004;52(2):89–101. https://doi.org/10.1016/j.plasmid.2004.05.002 PMID: 15336487
37. Zhang S, Meyer R. The relaxosome protein MobC promotes conjugal plasmid mobilization by extending DNA strand separation to the nick site at the origin of transfer. Mol Microbiol. 1997;25(3):509–16. https://doi.org/10.1046/j.1365-2958.1997.4861849.x PMID: 9302013
38. Harrison OB, Cehovin A, Skett J, Jolley KA, Massari P, Genco CA, et al. *Neisseria gonorrhoeae* Population Genomics: Use of the Gonococcal Core Genome to Improve Surveillance of Antimicrobial Resistance. J Infect Dis. 2020;222(11):1816–25. https://doi.org/10.1093/infdis/jiaa002 PMID: 32163580
39. San Millan A, MacLean RC. Fitness Costs of Plasmids: a Limit to Plasmid Transmission. Microbiol Spectr. 2017;5(5):10.1128/microbiolspec.mtbp-0016–2017. https://doi.org/10.1128/microbiolspec.MTBP-0016-2017 PMID: 28944751
40. Coluzzi C, Rocha EPC. The Spread of Antibiotic Resistance Is Driven by Plasmids Among the Fastest Evolving and of Broadest Host Range. Mol Biol Evol. 2025;42(3):msaf060. https://doi.org/10.1093/molbev/msaf060 PMID: 40098486
41. Kunimoto DY, Plummer FA, Namaara W, D'Costa LJ, Ndinya-Achola JO, Ronald AR. Urethral infection with Haemophilus ducreyi in men. Sex Transm Dis. 1988;15(1):37–9. https://doi.org/10.1097/00007435-198801000-00009 PMID: 3258676
42. Barza M, Weinstein L. Pharmacokinetics of the penicillins in man. Clin Pharmacokinet. 1976;1(4):297–308.
43. Retchless AC, Kretz CB, Chang H-Y, Bazan JA, Abrams AJ, Norris Turner A, et al. Expansion of a urethritis-associated Neisseria meningitidis clade in the United States with concurrent acquisition of N. gonorrhoeae alleles. BMC Genomics. 2018;19(1):176. https://doi.org/10.1186/s12864-018-4560-x PMID: 29499642
44. Bazan JA, Tzeng Y-L, Bischof KM, Satola SW, Stephens DS, Edwards JL, et al. Antibiotic Susceptibility Profile for the US Neisseria meningitidis Urethritis Clade. Open Forum Infect Dis. 2023;10(1):ofac661. https://doi.org/10.1093/ofid/ofac661 PMID: 36655188
45. Shen Z, Tang CM, Liu G-Y. Towards a better understanding of antimicrobial resistance dissemination: what can be learnt from studying model conjugative plasmids?. Mil Med Res. 2022;9(1):3. https://doi.org/10.1186/s40779-021-00362-z PMID: 35012680
46. Pagotto F, Dillon JA. Multiple origins and replication proteins influence biological properties of beta-lactamase-producing plasmids from *Neisseria gonorrhoeae*. J Bacteriol. 2001;183(19):5472–81. https://doi.org/10.1128/JB.183.19.5472-5481.2001 PMID: 11544207
47. Qin X, Chen Y, Wu X, Chen W, Xue Y, Huang J. Emerging epidemic of the Africa-type plasmid in penicillinase-producing Neisseria gonorrhoeae in Guangdong, China, 2013-2022. Emerg Microbes Infect. 2025;14(1):2440489.
48. Peters RPH, Garrett N, Chandiwana N, Kularatne R, Brink AJ, Cohen K, et al. Erratum: Southern African HIV Clinicians Society 2022 guideline for the management of sexually transmitted infections: Moving towards best practice. South Afr J HIV Med. 2022;23(1):1465. https://doi.org/10.4102/sajhivmed.v23i1.1465 PMID: 36479415

49. Caméléna F, Mérimèche M, Brousseau J, Mainardis M, Verger P, Le Risbé C, et al. Emergence of Extensively Drug-Resistant Neisseria gonorrhoeae, France, 2023. Emerg Infect Dis. 2024;30(9):1903–6. https://doi.org/10.3201/eid3009.240557 PMID: 39084693
50. Kozlov AM, Darriba D, Flouri T, Morel B, Stamatakis A. RAxML-NG: a fast, scalable and user-friendly tool for maximum likelihood phylogenetic inference. Bioinformatics. 2019;35(21):4453–5. https://doi.org/10.1093/bioinformatics/btz305 PMID: 31070718
51. Paradis E, Schliep K. ape 5.0: an environment for modern phylogenetics and evolutionary analyses in R. Bioinformatics. 2019;35(3):526–8. https://doi.org/10.1093/bioinformatics/bty633 PMID: 30016406
52. Yu G. Using ggtree to Visualize Data on Tree-Like Structures. Curr Protoc Bioinformatics. 2020;69(1):e96. https://doi.org/10.1002/cpbi.96 PMID: 32162851
53. Lam Gy-Y. Ggtree: an R package for visualization and annotation of phylogenetic tree with different types of meta-data. Methods Ecol Evol. 2019.
54. Abramson J, Adler J, Dunger J, Evans R, Green T, Pritzel A, et al. Accurate structure prediction of biomolecular interactions with AlphaFold 3. Nature. 2024;630(8016):493–500. https://doi.org/10.1038/s41586-024-07487-w PMID: 38718835
55. DeLano W. The PyMOL molecular graphics system. 2002. http://www.pymol.org/
56. Jurrus E, Engel D, Star K, Monson K, Brandi J, Felberg LE, et al. Improvements to the APBS biomolecular solvation software suite. Protein Sci. 2018;27(1):112–28. https://doi.org/10.1002/pro.3280 PMID: 28836357
57. Dillard JP. Genetic Manipulation of *Neisseria gonorrhoeae*. Curr Protoc Microbiol. 2011;Chapter 4:Unit4A.2. https://doi.org/10.1002/9780471729259.mc04a02s23 PMID: 22045584
58. Madeira F, Pearce M, Tivey ARN, Basutkar P, Lee J, Edbali O, et al. Search and sequence analysis tools services from EMBL-EBI in 2022. Nucleic Acids Res. 2022;50(W1):W276–9. https://doi.org/10.1093/nar/gkac240 PMID: 35412617
59. Watson JF, García-Nafría J. In vivo DNA assembly using common laboratory bacteria: A re-emerging tool to simplify molecular cloning. J Biol Chem. 2019;294(42):15271–81. https://doi.org/10.1074/jbc.REV119.009109 PMID: 31522138
60. Cartwright CP, Stock F, Gill VJ. Improved enrichment broth for cultivation of fastidious organisms. J Clin Microbiol. 1994;32(7):1825–6.
61. Unemo MR, Ison C, Lewis D, Ndowa F, Peeling R. Laboratory diagnosis of sexually transmitted infections, including human immunodeficiency virus. World Health Organization; 2013. https://iris.who.int/bitstream/handle/10665/85343/9789241505840_eng.pdf?sequence=1
62. Hechard T, Wang H. Determination of growth rate and virulence plasmid copy number during Yersinia pseudotuberculosis infection using droplet digital PCR. Methods Mol Biol. 2023;2674:101–15.
63. Wickham H, Averick M, Bryan J, Chang W, McGowan L, François R, et al. Welcome to the Tidyverse. JOSS. 2019;4(43):1686. https://doi.org/10.21105/joss.01686
64. Wickham H. Elegant graphics for data analysis. 2 ed. New York: Springer; 2016.
65. Zhou Z, Alikhan N-F, Sergeant MJ, Luhmann N, Vaz C, Francisco AP, et al. GrapeTree: visualization of core genomic relationships among 100,000 bacterial pathogens. Genome Res. 2018;28(9):1395–404. https://doi.org/10.1101/gr.232397.117 PMID: 30049790
66. Papadopoulos JS, Agarwala R. COBALT: constraint-based alignment tool for multiple protein sequences. Bioinformatics. 2007;23(9):1073–9. https://doi.org/10.1093/bioinformatics/btm076 PMID: 17332019
67. Robert X, Gouet P. Deciphering key features in protein structures with the new ENDscript server. Nucleic Acids Res. 2014;42(Web Server issue):W320-4. https://doi.org/10.1093/nar/gku316 PMID: 24753421
